# Effectiveness of social skills intervention for the management of children with autism spectrum disorder: A protocol for systematic review and meta-analysis

**DOI:** 10.1097/MD.0000000000020331

**Published:** 2020-05-29

**Authors:** Fen-Rong Shuai, Zhan-Yuan Lin

**Affiliations:** a Department of Pediatrics, Yan’an People's Hospital, Yan’an; b Department of Pediatrics, Yangling Demonstration Zone Hospital, Xianyang, China.

**Keywords:** autism spectrum disorder, effectiveness, safety, social skills intervention

## Abstract

**Background:**

This study will investigate the effectiveness of and safety of social skills intervention (SSI) for the management of children with autism spectrum disorder (ASD).

**Methods:**

All potential randomized controlled trials related to the effectiveness and safety of SSI for children with ASD will be retrieved from Cochrane Library, MEDLINE, EMBASE, Chinese Biomedical Literature Database, and China National Knowledge Infrastructure. All these databases will be identified from inception to the present with no limitations of language and publication time. Two investigators will independently perform selection of study, data collection, and study quality assessment, respectively. A third investigator will help to solve any different views between 2 investigators. RevMan 5.3 software will be used for data pooling and statistical analysis.

**Results:**

This study will provide synthesis of present evidence on assessing the effectiveness and safety of SSI for children with ASD.

**Conclusion:**

This study will provide helpful references for the effectiveness and safety of SSI on the management of ASD, which may benefit both patients and clinicians.

**Study registration number:**

INPLASY202040090.

## 1 Introduction

Autism spectrum disorder (ASD) is one of the serious neurodevelopmental disorders.^[1–4]^ It is characterized by impairments of communications and social interactive ability.^[5–7]^ It manifests as the presence of restricted and repetitive patterns of behaviors and interests.^[8–10]^ It has been estimated that its incidence rate is about 2 per 1000 children aged 8 years, with 23.6 per 1000 boys and 5.3 per 1000 girls.^[11]^ Thus, it is very important to manage ASC as early as possible. Previous studies have reported that social skills intervention (SSI) can effectively manage children with ASD.^[12–18]^ However, no systematic review focused on the SSI for children with ASD has been published. Thus, this study aims to assess the effectiveness and safety of SSI on the management of ASD.

## 2 Methods

### 2.1 Study registration

This study has been registered prospectively on INPLASY202040090. We report this study according to the Preferred Reporting Items for Systematic Reviews and Meta-Analyses Protocol 2015 statement.^[19]^

### 2.2 Eligibility criteria

#### 2.2.1 Type of studies

All randomized controlled trials that assess the use of SSI in the treatment of children with ASD will be included. However, we will exclude any other studies, such as animal studies, and non-clinical studies.

#### 2.2.2 Type of participants

All children (less than 18 years old) with ASD will be included in analysis, regardless their ethnicity, gender, and economic status.

#### 2.2.3 Type of interventions

Any forms of SSI utilized for the treatment of children with ASD will be included as an experimental group.

Any other interventions, except SSI used to treat children with ASD will be set as a control group in this study.

#### 2.2.4 Type of outcomes

The primary outcomes are adaptive behaviors, as measured by Vineland Adaptive Behavior Scale, or other relevant scales.

The secondary outcomes are motor skills, visual reception, communication, and language, as assessed by Mullen Scales for Early Learning or others; joint attention, joint engagement, repetitive behaviors, social skills, as checked by Autism Diagnostic Observation Schedule or other associated tools; child anxiety, child distress, parental distress, parental self-efficacy, parent child relationship, self-regulation, and symptom severity, as identified using Aberrant Behavior Checklist, or Autism Treatment Evaluation Checklist, Social Communication Questionnaire and Autism Behavior Checklist, or other scales; and any adverse events.

### 2.3 Search strategy

We will search all related randomized controlled trials on assessing effectiveness and safety of SSI for children with ASD from Cochrane Library, MEDLINE, EMBASE, Chinese Biomedical Literature Database, and China National Knowledge Infrastructure from inception to the present with no limitations of language and publication time. Search terms will include autism, ASD, Asperger, childhood disintegrative disorder, difficulty with communication, social interactions, imagination, creativity, obsessive interests, repetitive behaviors, social skills training, and education. A search strategy example of MEDLINE is presented in Table 1. We will also adapt similar search strategies to the other electronic databases.

**Table 1:**
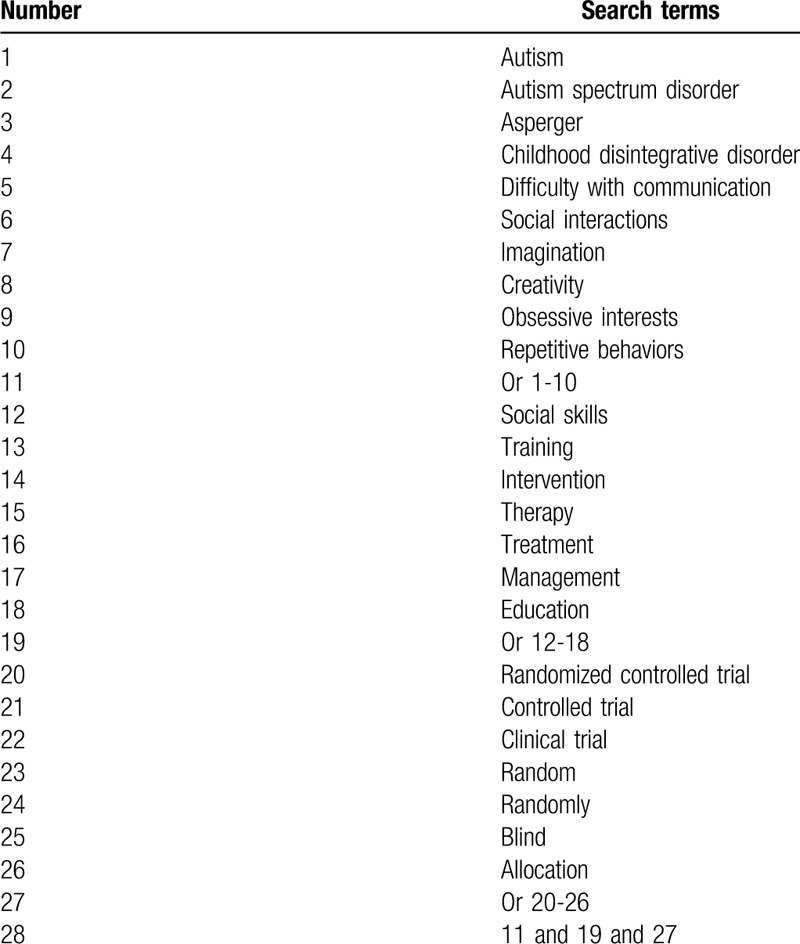
Search strategy for MEDLINE.

In addition, any other potential studies will be searched from relevant conference proceedings, dissertations, and reference lists of associated reviews.

### 2.4 Data selection

#### 2.4.1 Study selection

**Figure 1:**
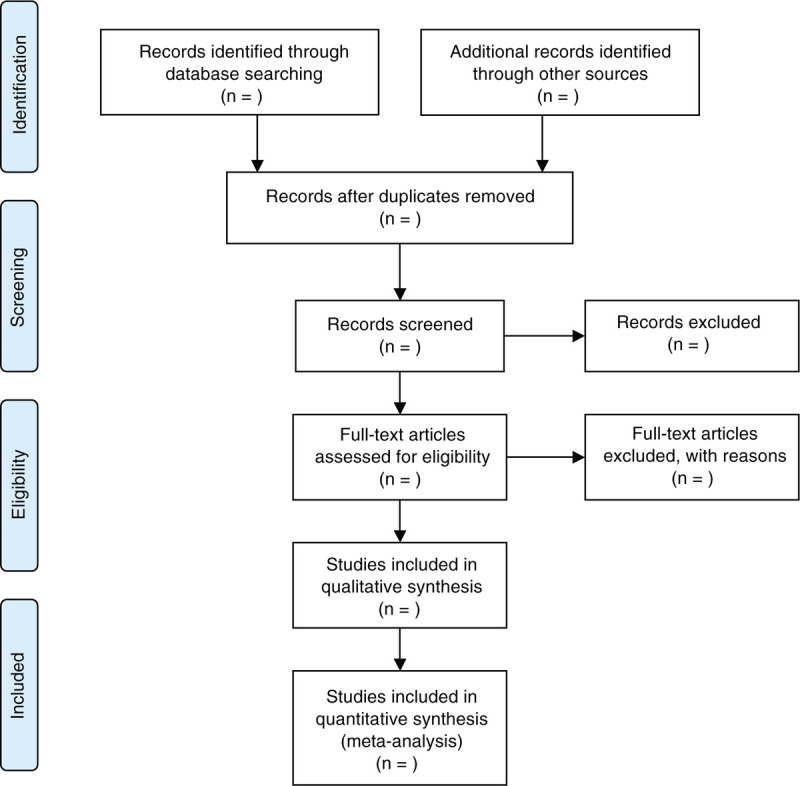
Flowchart of study selection.

Two investigators will independently undertake study selection, respectively according to the pre-designed eligibility criteria. They will choose the literature by scanning titles and abstracts of all searched records. The full text of remaining relevant papers will be carefully read to identify if they meet all inclusion criteria. We will note reasons for all excluded studies. Any disagreements regarding selection of study between 2 investigators will be resolved by a third investigator through discussion. The process of study selection will be shown in the flowchart (Fig. 1).

#### 2.4.2 Data extraction

Two investigators will independently collect data utilizing a previous designed data extraction sheet. Any discrepancies between 2 investigators will be solved by a third investigator via consultation. The extracted information includes study characteristics (such as title, first author, and time of publication), patient characteristics (such as age, gender, and diagnostic criteria), study setting, study methods (such as details of randomization, and blind), treatment and control details (such as management types, dosage, and frequency), outcomes, and conflicts of interest. When there are inadequate or missing data, we will attempt to contact primary corresponding authors by email or telephone to obtain such information.

### 2.5 Study quality assessment

Two investigators will independently assess the study quality using Cochrane Risk of Bias Tool based on the 7 domains, and each one is further assessed as low, unclear or high risk of bias. Any disagreements between two investigators will be solved by a third investigator.

### 2.6 Data analysis

RevMan 5.3 software will be applied for statistical analysis according to the homogeneity among eligible studies. Mean difference or standardized mean difference and 95% confidence intervals (CIs) will calculate continuous data, while risk ratio and 95% CIs will express dichotomous data. The *I*^*2*^ statistic will be used for checking heterogeneity among included studies. *I*^*2*^ ≤ 50 means homogeneity, while *I*^*2*^ > 50% means significant heterogeneity. If *I*^*2*^ ≤ 50, a fixed-effects model will be used, and meta-analysis will be performed if sufficient studies are included which focus on the similar study information, patient characteristics, interventions, controls and outcomes. Conversely, if *I*^*2*^ > 50%, a random-effects model will be applied, and subgroup analysis will be conducted to explore the potential reasons for the significant heterogeneity.

### 2.7 Subgroup analysis

If we detected significant heterogeneity among included studies, subgroup analysis will be carried out according to the different characteristics of study and patient, interventions, comparators, and outcomes.

### 2.8 Sensitivity analysis

If sufficient data are available, we will conduct sensitivity analysis to determine whether the merged outcome results are robust by excluding studies with high risk of bias.

### 2.9 Reporting bias

If a sufficient number of eligible studies are included, we will perform Funnel plot^[20]^ and Egger regression test^[21]^ to check potential reporting bias.

### 2.10 Ethics and dissemination

No individual patient data is required in this study, thus, no ethic approval is needed. We will publish this study on a peer-reviewed journal or a relevant conference meeting.

## 3 Discussion

Autism spectrum disorder is very common neurodevelopmental disease among children population. Although several medications are reported to manage this condition, their efficacy is still limited, and they also accompany a variety of adverse events. Thus, alternative interventions with fewer or no adverse events are urgently needed to treat such condition. Previous studies have reported that SSI can greatly benefit children with ASD with fewer or no adverse events. Unfortunately, its efficacy is still inconsistent, and no systematic review has been conducted to explore this issue. Therefore, this study will comprehensively search related literatures, and will systematically assess the effectiveness and safety of SSI for the children with ASD. The results of this study will summarize the most recent evidence of SSI for ASD and may provide helpful evidence for the clinical practice.

## Author contributions

**Conceptualization:** Fen-Rong Shuai, Zhan-yuan Lin.

**Data curation:** Fen-Rong Shuai, Zhan-Yuan Lin.

**Formal analysis:** Fen-Rong Shuai, Zhan-Yuan Lin.

**Investigation:** Zhan-Yuan Lin.

**Methodology:** Fen-Rong Shuai.

**Project administration:** Zhan-Yuan Lin.

**Resources:** Fen-Rong Shuai.

**Software:** Fen-Rong Shuai.

**Supervision:** Zhan-Yuan Lin.

**Validation:** Fen-Rong Shuai, Zhan-Yuan Lin.

**Visualization:** Fen-Rong Shuai, Zhan-Yuan Lin.

**Writing – original draft:** Fen-Rong Shuai, Zhan-Yuan Lin.

**Writing – review & editing:** Fen-Rong Shuai, Zhan-Yuan Lin.
